# Carrageenophyte *Kappaphycus malesianus* Inhibits Microglia-Mediated Neuroinflammation via Suppression of AKT/NF-*κ*B and ERK Signaling Pathways

**DOI:** 10.3390/md20080534

**Published:** 2022-08-20

**Authors:** Nicole Jean-Yean Lai, Ee-Ling Ngu, Jun-Rui Pang, Kah-Hui Wong, Chrismawan Ardianto, Long Chiau Ming, Siew-Huah Lim, Shweta Gangasa Walvekar, Ayaz Anwar, Yoon-Yen Yow

**Affiliations:** 1 Department of Biological Sciences, School of Medical and Life Sciences, Sunway University, Bandar Sunway 47500, Malaysia; 2 Department of Anatomy, Faculty of Medicine, Universiti Malaya, Kuala Lumpur 50603, Malaysia; 3 Department of Pharmacy Practice, Faculty of Pharmacy, Universitas Airlangga, Surabaya 60115, Indonesia; 4 PAPRSB Institute of Health Sciences, Universiti Brunei Darussalam, Gadong BE1410, Brunei; 5 Department of Chemistry, Faculty of Science, Universiti Malaya, Kuala Lumpur 50603, Malaysia

**Keywords:** red seaweed, BV2 microglia, antineuroinflammatory, neuroprotective, proinflammatory cytokines, local breeds, sustainable

## Abstract

Neuroinflammation is an inflammatory response in any part of the central nervous system triggered by the activation of microglia and astrocytes to produce proinflammatory cytokines in the brain. However, overproduction of proinflammatory cytokines further contributes to the development of neurodegenerative disorders. Red seaweed, *Kappaphycus malesianus,* is a predominant carrageenophyte commercially cultivated in Semporna, Sabah, Malaysia. It is an important source of raw material for kappa-carrageenan productions in the food, pharmaceutical and cosmetics industries. However, no studies have been conducted focusing on the antineuroinflammatory effects of *K. malesianus*. The aim of the present study was to investigate the effect of the antineuroinflammatory activity of *K. malesianus* extracts (ethyl acetate, ethanol and methanol) on lipopolysaccharide-stimulated BV2 microglia and the underlying mechanisms involved in the regulation of neuroinflammatory pathways. Extract with the most promising antineuroinflammatory activity was analyzed using liquid chromatography-mass spectrometry (LC-MS). Our results show that methanol extract has a convincing antineuroinflammatory effect by suppressing both AKT/NF-*κ*B and ERK signaling pathways to inhibit the expression of all proinflammatory cytokines without causing a cytotoxicity effect. LC-MS analysis of methanol extract revealed two compounds: prosopinine and eplerenone. Our findings indicated that metabolites of *K. malesianus* are potent antineuroinflammatory agents with respect to prevention of neurological disorders.

## 1. Introduction

The global average life expectancy has increased due to advanced medical treatments and technologies. According to Our World in Data, the median age of the global population has increased from 21.5 years to 30 years in the past 49 years [1]. However, population aging has led to a steep increase in age-related diseases, such as dementia. Research by Feigin et al. on the global burden of diseases, injuries, and risk factors (GDB) estimated that there are almost 44 million people affected by dementia globally, which has more than doubled since 1990 [2]. Neurodegenerative diseases are one among the most common causes of dementia, including Alzheimer’s, Parkinson’s and Huntington’s diseases, as well as several types of multiple sclerosis [3]. As there is no disease-modifying treatment for these disorders, our current interest is various seaweed-derived phytochemicals as potential treatment options [4].

Chronic neuroinflammation is closely related to neurodegenerative diseases. The persistent production of proinflammatory mediators or cytokines, such as nitric oxide (NO), cyclooxygenase-2 (COX-2), tumor necrosis factor (TNF)-α, interleukin (IL)-1 and IL-6, exacerbates neuroinflammation, causing neuronal damage and further leading to neurodegenerative diseases [5,6]. The expression of these proinflammatory mediators or cytokines is regulated by AKT and NF-*κ*B signaling pathways [7,8]. In addition, the AKT signaling pathway plays a major role in regulating GSK-3β activity in cells, as abnormal activity of GSK-3β could result in disorientated production of neuronal proteins, which can also trigger neuroinflammation [9,10,11]. NF-*κ*B is an inducible transcriptional factor that carries a transduction signals between the cytoplasm and nucleus [12]. As such, activation of NF-*κ*B by lipopolysaccharide (LPS) triggers a signaling cascade to produce proinflammatory mediators [13,14].

Seaweeds are well known as nutraceutical or functional food due to their medicinal and therapeutic properties. Secondary metabolites from seaweeds promote growth rate of tilapia fish [15,16], improve non-specific immune response [16] and contribute to pharmacological activities, such as antioxidant [17,18,19], antiinflammatory [20], antimicrobial [19,21] and antifungal [22] activities. Bioactive compounds such as fucoxanthin and sargachromenol from brown seaweeds (*Sargassum* spp.) [23], honaucins A–C from *Leptolyngbya crossbyana* [24] and sacran from *Aphanothece sacrum* [25] have been reported to exhibit antiinflammatory activities. *Kappaphycus malesianus*, also known as “*Aring-aring*”, was identified as the new member of *Kappaphycus* family in 2014 and displays considerable morphological similarity with *K. alvarezii* [26]. *K. malesianus* is a red seaweed widely cultivated in Semporna, Sabah, Malaysia, as an important source of raw material for carrageenan production for use as an emulsifier and stabilizer in the food, pharmaceutical and cosmetics industries. *K. alvarezii* has been reported to exhibits antiinflammatory activities and neuroprotective activities by promoting neuronal growth, which could slow down the process of aging [27,28]. As *K. malesianus* and *K. alvarezii* are from the same family, are usually cultivated together and share similar morphology, it is hypothesized that *K. malesianus* extract could potentially exert an antiinflammatory effect. To the best of our knowledge, the pharmacological properties of *K. malesianus* are largely unexplored; hence, the aim of this project was to investigate the antineuroinflammatory activity of *K. malesianus* extract on LPS-stimulated BV2 microglia and the underlying mechanism of this action. The possible compounds present in the extract with the most promising antineuroinflammatory activity were identified by liquid chromatography–mass spectrometric (LC-MS) analysis.

## 2. Results

### 2.1. Effect of K. malesianus Extracts on the Viability of BV2 Microglia

**Figure 1:**
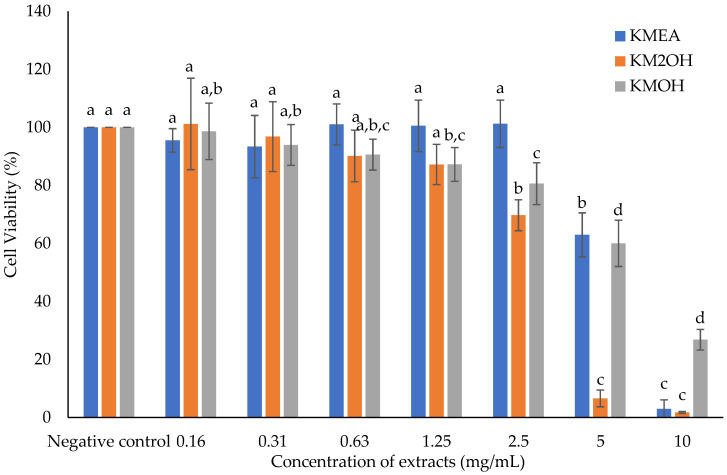
The effect of ethyl acetate, ethanol and methanol extracts of *K. malesianus* on cell viability of BV2 microglia evaluated by MTT assay. Different letters on top of the bars indicated significant differences (*p* ≤ 0.05, one-way ANOVA: Duncan test). All data are shown as the mean ± SD in triplicate (*n* = 3). KMEA: ethyl acetate extract; KM2OH: ethanol extract; KMOH: methanol extract.

We investigated the cell viability of BV2 microglia treated with various *K.* *malesianus* extracts (ethyl acetate, ethanol and methanol extracts) at concentrations ranging from 0 mg/mL to 10 mg/mL, with a negative control containing BV2 microglia and media only (Figure 1).

Figure 1 shows that cell viability of BV2 microglia treated with *K. malesianus* ethyl acetate extract stayed above 90% from 0 mg/mL to 2.5 mg/mL; however, it significantly decreased (*p* ≤ 0.05) to 62.95% ± 7.59 and 2.98% ± 3.12 at 5 mg/mL and 10 mg/mL, respectively, compared to the negative control (*p* ≤ 0.05). Results show that cell viability of BV2 microglia treated with *K. malesianus* ethanol extract gradually decreased from 0.16 mg/mL (101.19% ± 15.75) to 2.5 mg/mL (69.74% ± 5.33), followed by a sharp declined at 5 mg/mL (6.58% ± 2.87). We also found that cell viability of BV2 microglia treated with methanol extract gradually decreased until 5 mg/mL (60.03% ± 7.97), with a sudden decline in cell viability at 10 mg/mL (26.83% ± 3.55). We observed a significant decrease (*p* ≤ 0.05) in cell viability of BV2 microglia treated with all *K. malesianus* extracts, starting from 5 mg/mL; therefore, in the subsequent bioassays, the concentration of *K. malesianus* extracts was maintained below a maximum of 2.5 mg/mL.

### 2.2. Effect of K. malesianus Extracts on NO Production in LPS-Stimulated BV2 Microglia

The effect of *K. malesianus* extracts on NO inhibition in the supernatant media of BV2 microglia was determined after the cells were treated with 1 µg/mL LPS with concentrations of *K. malesianus* extracts ranging from 0.16 mg/mL to 2.5 mg/mL. The three control groups were untreated control (only media), negative control (media and LPS) and positive control (N(γ)-nitro-L-arginine methyl ester (L-NAME) and LPS).

**Figure 2:**
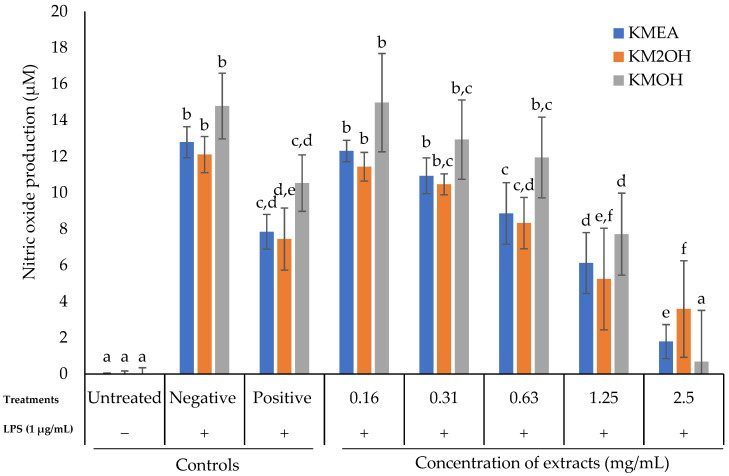
Nitric oxide production of LPS-stimulated BV2 microglia against various concentrations of ethyl acetate, ethanol and methanol extracts of *K. malesianus* evaluated by Griess assay. Different letters on top of the bars indicated significant differences (*p* ≤ 0.05, one-way ANOVA: Duncan test). All data are shown as the mean ± SD in triplicate (*n* = 3).

As shown in Figure 2, ethyl acetate extract gradually inhibited NO production as the concentration increased. At 2.5 mg/mL, NO production was inhibited to 1.79 µM ± 0.94. Starting from the concentration of 0.63 mg/mL (8.87 µM ± 1.7), a significant difference (*p* ≤ 0.05) relative to the negative control (12.79 µM ± 0.85) was observed, whereas the NO inhibitory activity was comparable to that of the positive control (7.85 µM ± 0.96). For ethanol extract, a gradual decrease in NO production was observed until a sudden decline to 3.59 µM ± 2.66 at 2.5 mg/mL. NO production of ethanol extract was significantly lower (*p* ≤ 0.05) than in the negative control starting from 0.63 mg/mL, comparable to the positive control. *K. malesianus* methanol extract significantly (*p* ≤ 0.05) inhibited NO production to 0.68 µM ± 2.83 at a concentration of 2.5 mg/mL, comparable to the untreated control. *K. malesianus* methanol extract showed the highest NO inhibitory activity among all extracts, as it reduced the NO production to 0.68 mg/mL. Moreover, *K. malesianus* methanol extract at 2.5 mg/mL showed no significant difference (*p* ≥ 0.05) relative to the untreated control, indicating that methanol extract at 2.5 mg/mL was capable of reducing NO production to the normal state, like the untreated control. Hence, methanol extract was chosen for the subsequent experiments, as it showed the most potent NO inhibitory activity in LPS-stimulated BV2 microglia.

### 2.3. Effects of K. malesianus Methanol Extract on iNOS and COX-2 Protein Expression in LPS-Stimulated BV2 Microglia

**Figure 3:**
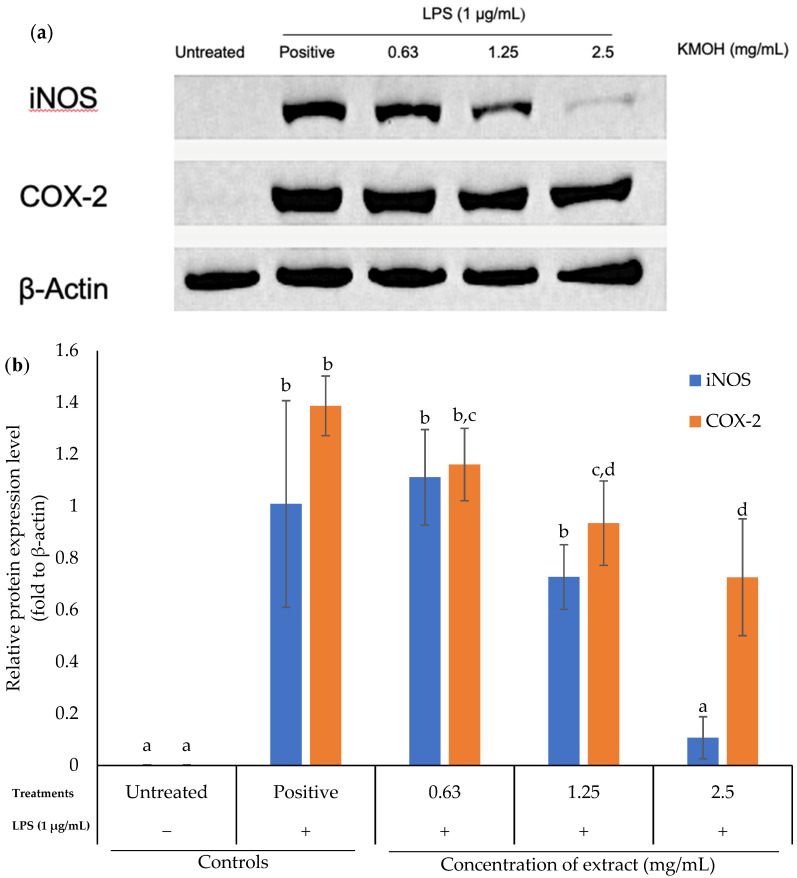
(**a**) Effects of *K. malesianus* methanol extract in LPS-stimulated BV2 microglia on iNOS and COX-2 expression, with β-actin as the housekeeping gene. (**b**) Relative protein expression levels of iNOS and COX-2 were determined by densitometry and normalized by β-actin. Different letters on top of the bars indicated significant differences (*p* ≤ 0.05, one-way ANOVA: Duncan test). All data are shown as the mean ± SD in triplicate (*n* = 3).

Effects of *K. malesianus* methanol extract on proinflammatory mediators (iNOS and COX-2) with β-actin as the housekeeping gene are shown in Figure 3. The tested concentrations of *K. malesianus* methanol extract ranged from 0.63 mg/mL to 2.5 mg/mL, with two control groups: untreated control (only media) and positive control (media and LPS). Methanol extract decreased the LPS-stimulated expression of both iNOS and COX-2, with a particularly obvious dose-dependent reduction in iNOS. β-actin bands were similar between the control and treatment groups. According to protein quantification results (Figure 3b), treatment with methanol extract dose-dependently downregulated the expression of iNOS and COX-2 proteins. At concentrations between 1.25 mg/mL and 2.5 mg/mL, the expression of iNOS and COX-2 was lower than that of the positive control, showing the antineuroinflammatory activity of *K. malesianus* methanol extract.

### 2.4. Effect of K. malesianus Methanol Extract on Proinflammatory Cytokines Expression in LPS-Stimulated BV2 Microglia

**Figure 4:**
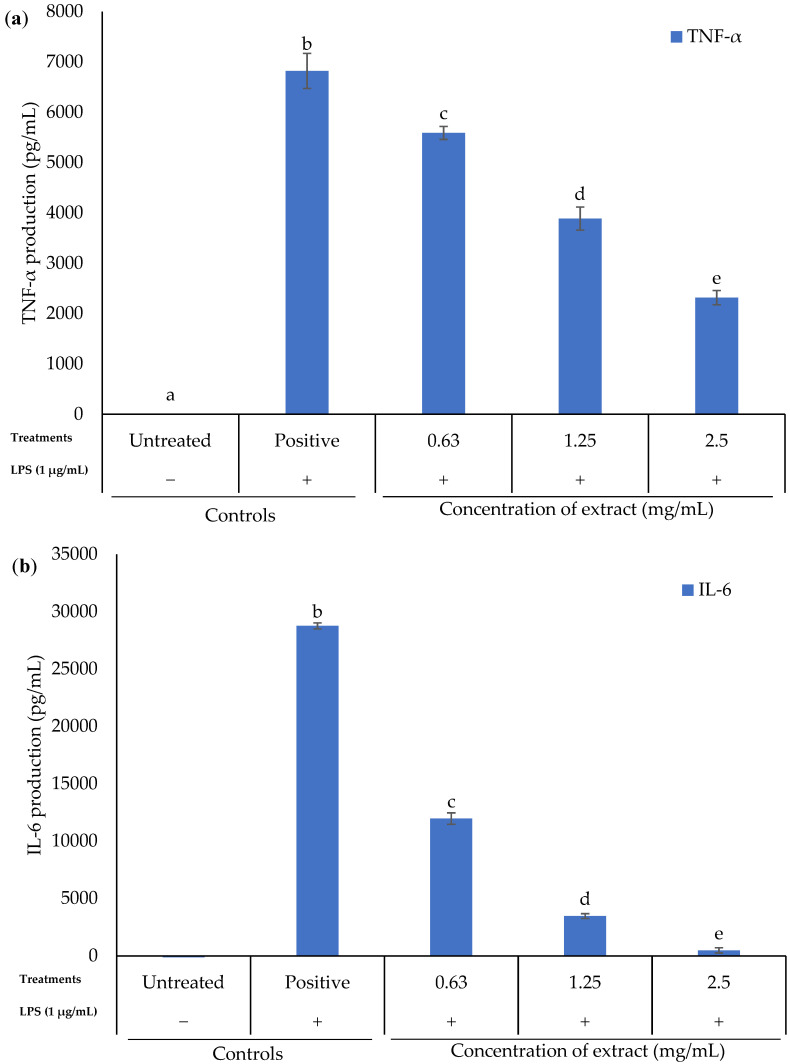
(**a**) TNF-α and (**b**) IL-6 production of LPS-stimulated BV2 microglia against various concentrations of methanol extract of *K. malesianus* evaluated by ELISA kit. Different letters on top of the bars indicated significant differences (*p* ≤ 0.05, one-way ANOVA: Duncan test). All data are shown as the mean ± SD in triplicate (*n* = 3).

The expression of proinflammatory cytokines (TNF-α and IL-6) in LPS-stimulated BV2 microglia was evaluated using ELISA (Figure 4). The tested concentrations of *K. malesianus* methanol extract ranged from 0.63 mg/mL to 2.5 mg/mL, with two control groups: untreated control (only media) and positive control (media and LPS). Results showed that methanol extract significantly (*p* ≤ 0.05) downregulated the expression of TNF-α and IL-6 in LPS-stimulated BV2 microglia in a dose-dependent manner, with a higher inhibitory effect on IL-6. Therefore, pre-treatment with methanol extract could suppressed the expression of proinflammatory cytokines. Our findings further verified the antineuroinflammatory effect of *K. malesianus* methanol extract.

### 2.5. Effect of K. malesianus Methanol Extract on Proinflammatory Mediators in LPS-Stimulated BV2 Microglia Using RT-PCR

**Figure 5:**
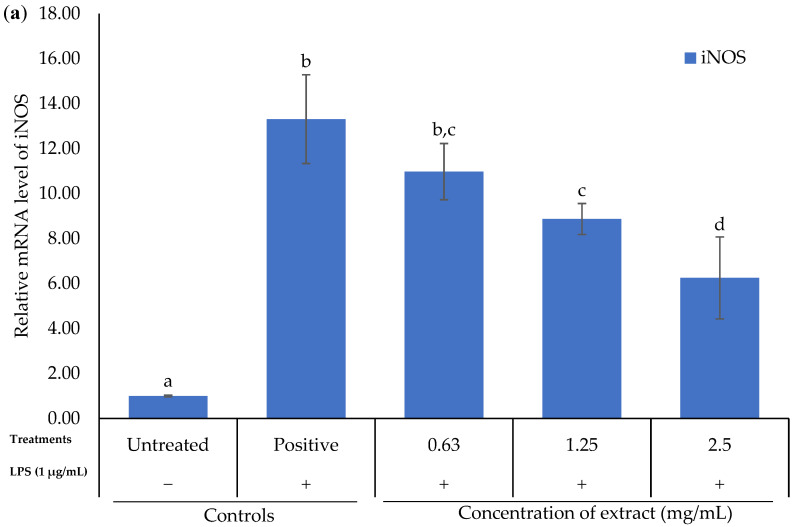

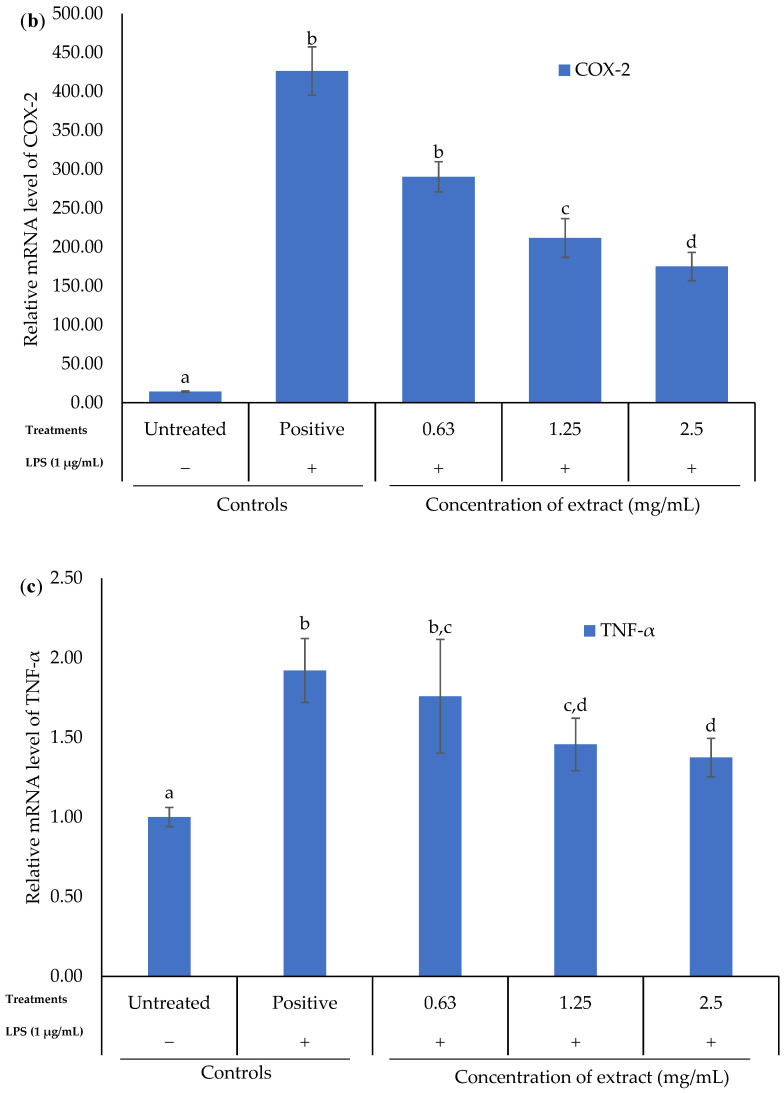

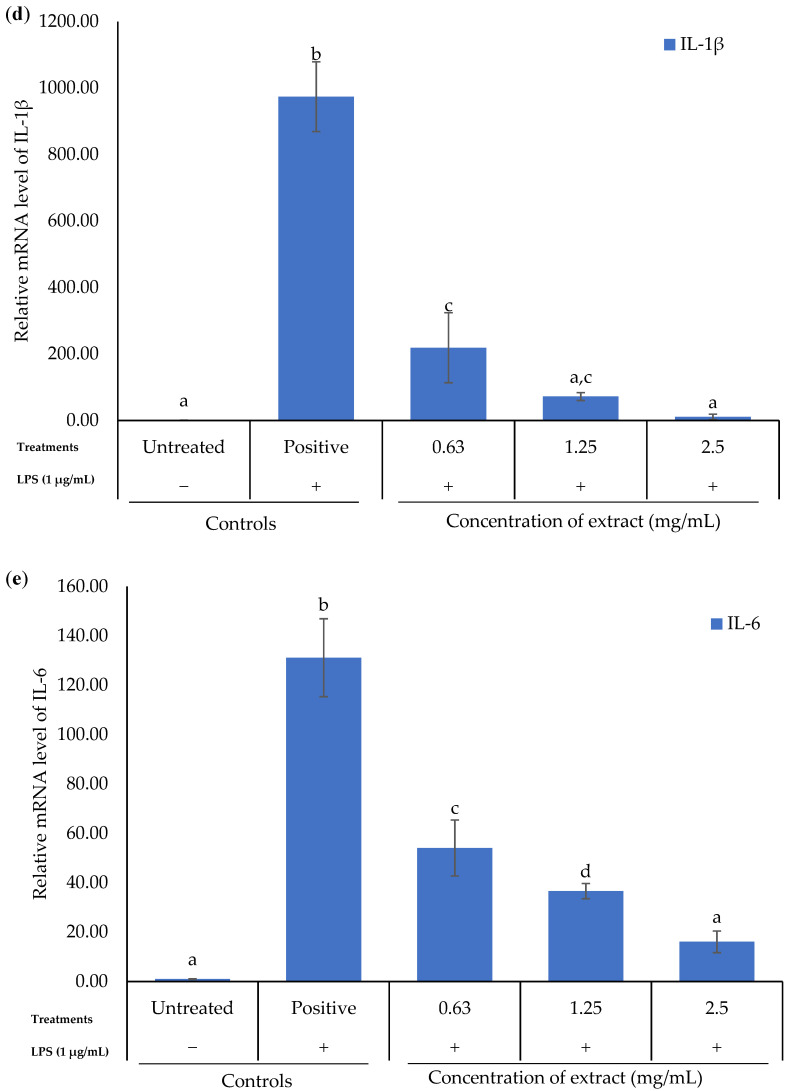
RT-PCR results of the effect of *K. malesianus* methanol extract on (**a**) iNOS, (**b**) COX-2, (**c**) TNF-α, (**d**) IL-1β and (**e**) IL-6 in LPS-stimulated BV2 microglia. Different letters on top of the bars indicated significant differences (*p* ≤ 0.05, one-way ANOVA: Duncan test). All data were normalized against a GAPDH control and are expressed as the mean ± SD in triplicate (*n* = 3).

RT-PCR was used to determine the mRNA level of proinflammatory mediators and cytokines (iNOS, COX-2, TNF-α, IL-1β and IL-6) in *K. malesianus* methanol extract-treated, LPS-stimulated BV2 microglia (Figure 5). The tested concentrations of methanol extract ranged from 0.63 mg/mL to 2.5 mg/mL, with two control groups: untreated control (only media) and positive control (media and LPS). Results indicated that the methanol extract inhibited the mRNA level of all mediators and cytokines in a dose-dependent manner as the extract concentrations increased. These results are consistent with the immunoblot results, suggesting that *K. malesianus* methanol extract has the potential to suppress the transcription of proinflammatory mediators and cytokines.

### 2.6. Effect of K. malesianus Methanol Extract on the AKT and ERK Signaling Pathway in LPS-Stimulated BV2 Microglia

**Figure 6:**
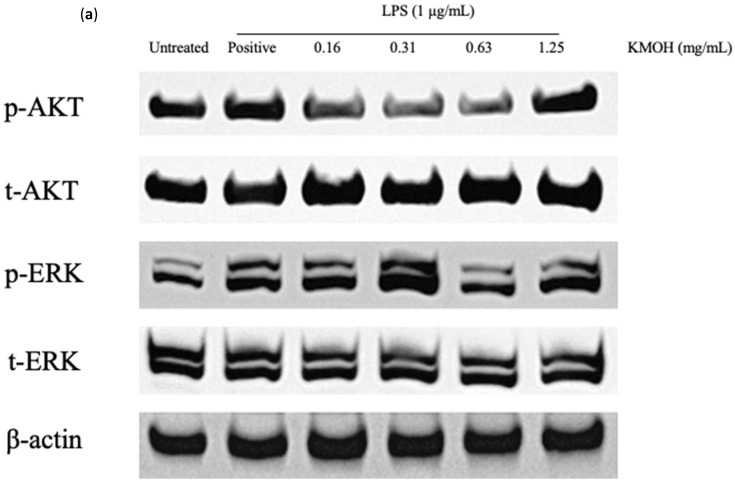

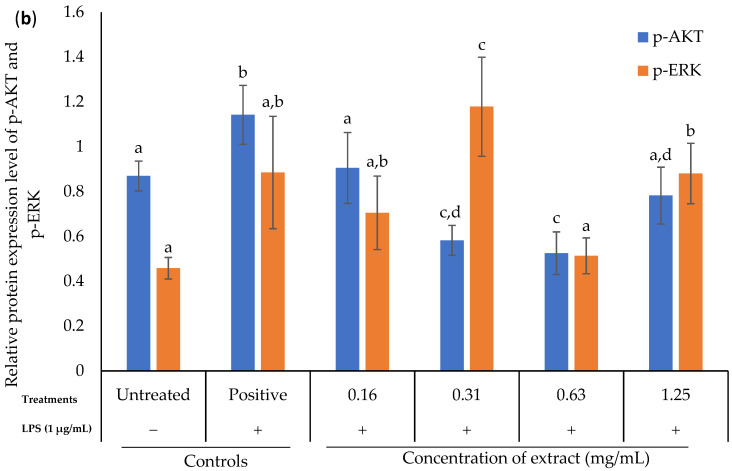
(**a**) Effects of *K. malesianus* methanol extract in LPS-stimulated BV2 microglia on p-AKT proteins, t-AKT proteins, p-ERK proteins and t-ERK proteins, with β-actin as the housekeeping gene. (**b**) The relative expression levels of p-AKT proteins and p-ERK proteins were determined by densitometry and normalized by t-AKT proteins and t-ERK proteins, respectively. Different letters on top of the bars indicated significant differences (*p* ≤ 0.05, one-way ANOVA: Duncan test). All data are shown as the mean ± SD in triplicate (*n* = 3).

*K. malesianus* methanol extract was used to further investigate the effects on signaling pathways, including the AKT and ERK pathways in LPS-stimulated BV2 microglia (Figure 6). Phosphorylated AKT (p-AKT) proteins and phosphorylated ERK (p-ERK) proteins were investigated with methanol extract at concentrations ranging from 0.16 mg/mL to 1.25 mg/mL, with two control groups: untreated control (only media) and positive control (media and LPS). Total AKT (t-AKT) proteins and total ERK (t-ERK) proteins were also observed to ensure the validity of results obtained from the phosphorylated proteins, with β-actin as the housekeeping gene. Results showed that methanol extract suppressed p-AKT proteins in a dose-dependent manner, with an optimal inhibitory activity at 0.63 mg/mL, followed by a sudden increase in protein expression at 1.25 mg/mL. On the other hand, p-ERK proteins showed upregulated expression from 0.16 mg/mL to 0.31 mg/mL, followed by a steep decline in protein expression at a concentration of 0.63 mg/mL and a slight increase at 1.25 mg/mL. Thus, 0.63 mg/mL of *K. malesianus* methanol extract is suggested as the optimal concentration for antineuroinflammatory activity.

### 2.7. Effect of K. malesianus Methanol Extract on the NF-κB Signaling Pathway in LPS-Stimulated BV2 Microglia

**Figure 7:**
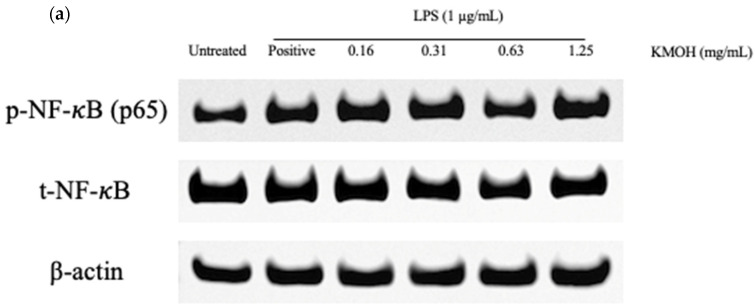

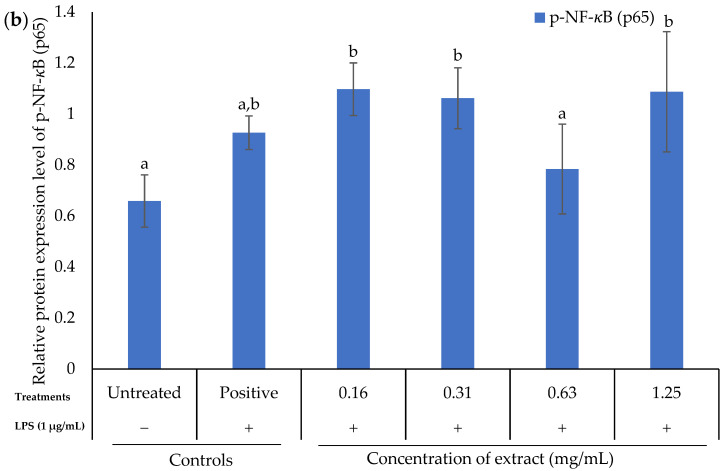
(**a**) Effects of *K. malesianus* methanol extract in LPS-stimulated BV2 microglia on p-NF-*κ*B proteins and t-NF-*κ*B proteins, with β-actin as the housekeeping gene. (**b**) The relative expression levels of p-NF-*κ*B proteins were determined by densitometry and normalized by t-NF-*κ*B proteins. Different letters on top of the bars indicated significant differences (*p* ≤ 0.05, one-way ANOVA: Duncan test). All data are shown as the mean ± SD in triplicate (*n* = 3).

Total protein of NF-*κ*B was extracted from both the cytoplasm and nucleus; the results (Figure 7) showed the effect of *K. malesianus* methanol extract on the expression of p-NF-*κ*B proteins in LPS-stimulated BV2 microglia at concentrations ranging from 0.16 mg/mL to 1.25 mg/mL, with two control groups: untreated control (only media) and positive control (media and LPS). Methanol extract gradually inhibited the expression of p-NF-*κ*B proteins, with a decline in inhibitory activities beginning at 1.25 mg/mL and the optimal inhibitory activity at 0.63 mg/mL.

### 2.8. Proposed Bioactive Compounds Present in K. malesianus Methanol Extract

LC-MS analysis of *K. malesianus* methanol extract detected 43 peaks in the positive-ion mass spectra. After comparison with 43 compounds, seven identified bioactive compounds were reported in the Metlin database, with molecular formula generator (MFG) scores above 90% and a ±2 difference in MFG scores. Among the seven identified bioactive compounds, only six (2,6-nonadien-1-ol, xestoaminol c, glutamyl-proline, prosopinine, 1-monopalmitin and eplerenone) had been reported with their bioactivities, including antitumor, antimicrobial, antiparasitic, anaesthetic, analgesic, antiviral and antiinflammatory activities. Table 1 showed the bioactive compounds identified in *K. malesianus* methanol extract.

## 3. Discussion

*K. malesianus* is a red seaweed discovered in 2014 and cultivated for carrageenan production. No pharmacological properties of this red seaweed have been investigated to date. Therefore, the present study is the first to report the antineuroinflammatory activity of *K. malesianus*.

We compared three solvent extracts—ethyl acetate, ethanol and methanol—and assessed the extracts’ cytotoxicity with respect to cell viability of BV2 microglia. *K. malesianus* methanol extract exhibited the least cytotoxicity compared to ethyl acetate and ethanol extracts. Methanol extract had an IC_50_ of 6.67 ± 0.61 mg/mL, indicating that 50% of the cell growth was inhibited at this concentration. ICH (International Council of Harmonisation of Technical Requirements for Pharmaceuticals for Human Use) guidelines suggest that products dosage less than 50 mg/mL containing organic solvents are safe for human consumption; thus, we suggest that our *K. malesianus* methanol extract is safe for use as alternative therapeutic agents [42]. Furthermore, our results demonstrated that methanol extract had the highest NO inhibitory activity among the tested extracts. Research studies have reported that ethanol and methanol are suitable to extract polyphenols and soluble phenolic compounds from plants due to the solubility of the bioactive compounds and polarity of the solvent [43,44]. Hence, ethanol and methanol are commonly used for the extraction of bioactive compounds, as most of the polyphenols and phenolic compounds possess antioxidant, antidiabetic and antiinflammatory activities [45,46,47]. In comparison with our results, *K. alvarezii* showed the highest NO inhibitory activity in ethyl acetate extract [48]. Although *K. malesianus* and *K. alvarezii* are often cultivated together and had similar morphology, it is believed that variation in bioactive compounds affect their bioactivities [26]. As such, it is believed that bioactive compounds in *K.* *malesianus* have higher polarity compared to those in *K. alvarezii*, causing varying effectiveness of NO inhibition in LPS-stimulated BV2 microglia. Our results showed that methanol is the best solvent for extracting bioactive compounds with antineuroinflammatory activity from *K. malesianus*. Therefore, methanol extract with the highest potency was chosen for further investigation of antineuroinflammatory activity.

As mentioned earlier, proinflammatory mediators are among the key factors that ameliorate neuroinflammation; therefore, modulation of the production of proinflammatory mediators is necessary to ease neuroinflammatory conditions. We found that *K. malesianus* effectively suppressed iNOS and COX-2 protein expressions. Similar results were shown in other red seaweeds, such as *Polyopes lancifolius* and *Laurencia snackeyi*, reducing the production of NO and PGE_2_ through the suppression of iNOS and COX-2 proteins in BV2 microglia and RAW 264.7 cells, respectively [49,50]. In addition, a study on the brown seaweed *Petalonia binghamiae* in Korea reported a similar pattern as that of *K. malesianus* methanol extract, whereby the band intensity decreased as the extract concentration increased [51]. Hence, a reduction in iNOS and COX-2 expression had proven the antineuroinflammatory activity of *K. malesianus* by suppressing both enzymes at the mRNA level. Moreover, *K. malesianus* methanol extract considerably reduced TNF-α and IL-6 production at the mRNA level. However, it was reported that methanol extract of the red seaweed *P. lancifolius* significantly inhibited TNF-α production at 0.1 mg/mL, with a greater inhibitory activity as compared to *K. malesianus*, which significantly inhibited TNF-α production at 1.25 mg/mL [49]. Purified terpenoid extract derived from crude methanol extract of *Hypnea musciformis* significantly reduced IL-6 production at 0.05 mg/mL as compared to *K. malesianus* crude methanol extract at 0.63 mg/mL [52]. In addition, a natural multimineral called Aquamin derived from red seaweed *Lithothamnion corallioides*, which has been approved by U.S. Food and Drug Administration (FDA) as a food supplement, was reported to exhibit antineuroinflammatory activity by suppressing the production of TNF-α and IL-1β [53,54]. Therefore, it is believed that our red seaweed, *K. malesianus*, exhibits antineuroinflammatory activity by regulating the expression of proinflammatory mediators and cytokines.

In our study, *K. malesianus* methanol extract exhibited antineuroinflammatory activity by blocking the signaling pathways, including AKT/NF-*κ*B and ERK pathways, thus suppressing the production of proinflammatory cytokines and contributing to the antineuroinflammatory activity. In 2005, it was found that AKT plays a role in regulating iNOS and COX-2 expression, as inhibiting AKT activation reduces the expression of iNOS and COX-2 [55]. Research revealed that seaweed-derived phenolic compounds are effective in inhibiting proinflammatory cytokines by regulating MAPK pathways, including ERK, thus suppressing inflammation [56]. Phosphorylation of I-*κ*Bα in NF-*κ*B is also one of the key factors that regulates the activation of iNOS and COX-2, as well as the production of TNF-α [57]. Our results showed that *K. malesianus* methanol extract was able to reduce the expression of NF-*κ*B gradually; therefore, it is strongly suggested that blocking the expression of NF-*κ*B could reduce the activation of proinflammatory mediators and cytokines. Collectively, our results suggested that *K. malesianus* exhibits antineuroinflammatory activity by regulating proinflammatory mediators and cytokines through the AKT/NF-*κ*B and ERK pathways. Moreover, we hypothesize that the reported bioactive compound(s) found in *K. malesianus* could contribute to antineuroinflammatory activity by blocking the AKT/NF-*κ*B and ERK pathways.

In the present study, among the seven reported bioactive compounds, including prosopinine and eplerenone, which had exhibited antiinflammatory activity, 2,6-nonadien-1-ol is predicted to have antiinflammatory activity. 2,6-nonadien-1-ol is a major aroma compound in black garlic, and research indicates that black garlic exhibits antiinflammatory activity by regulating the expression of NO, TNF-α and PGE_2_ [29,58]. Prosopinine is a piperidine alkaloid that was found to exhibit antibiotic, anaesthetic and analgesic properties [59]. Prosopinine found in *Prosopis africana* methanol stem bark extract was found to contribute to analgesic and antiinflammatory activities [35]. Eplerenone is a selective aldosterone blocker that can help to reduce the mortality rate of cardiovascular diseases [39]. Moreover, eplerenone has also been reported to have an antiinflammatory effect on viral myocarditis, suggesting that it could reduce inflammation during the development of heart failure [41]. According to our LC-MS results, 2,6-nonadien-1-ol was present in our *K. malesianus* methanol extract; thus, we predict that 2,6-nonadien-1-ol could contribute to the antiinflammatory activity in *K. malesianus*. However, in order to identify the role of 2,6-nonadien-1-ol in anti-inflammatory activity, further investigation is needed to justify this hypothesis. Lastly, prosopinine and eplerenone found in *K. malesianus* may contribute to antineuroinflammatory activity and have significant potential as antineuroinflammatory agents.

## 4. Materials and Methods

### 4.1. Seaweed Collection and Extract Preparation

Specimens of *K. malesianus* were collected from Semporna, Sabah, Malaysia. Herbarium voucher (KM_001) was prepared and deposited at Sunway University, Selangor Darul Ehsan, Malaysia. Specimens were washed with salt water to remove sand, mud and epiphytes, followed by a final rinsed with distilled water. Specimens were freeze-dried (LaboGene, Brigachtal, Germany) and ground into powder form before storage at −20 °C for future use. An amount of 5 g of seaweed powder was incubated with 250 mL of ethyl acetate, ethanol or methanol in a ratio of 1:50 (*w/v*) for 48 h at 37 °C in an incubator shaker, followed by centrifugation at 15,000 rpm for 10 min. All solvent extracts were vacuum-dried with a vacuum concentrator (LaboGene, Brigachtal, Germany) and stored at −20 °C for future use.

### 4.2. Cell Culture

Murine BV2 microglia (Elabscience, EP-CL-0493, Wuhan China) were cultured and maintained in Minimum Essential Medium Eagle (MEM) (Sigma-Aldrich, M0643, St. Louis, Mo, USA) supplemented with 10% fetal bovine serum (FBS) (Sigma-Aldrich, St. Louis, Mo, USA) and 1% penicillin-streptomycin (Sigma-Aldrich, St. Louis, Mo, USA) at 37 ± 2 °C in a 5% CO_2_-humidified incubator. The cells used in this study was controlled within passage numbers of 3–15.

### 4.3. 3-(4,5-dimethylthiazol-2yl)-2,5-diphenyl Tetrazolium Bromide (MTT) Cell Viability Assay

BV2 microglia were plated in a 96-well plate at a cell density of 6.25 × 10^4^ cells per well and incubated for 24 h. Then, the cells were treated with ethyl acetate, ethanol or methanol extract in a 2-fold dilution for another 24 h. On the third day, 10 μL of MTT (Merck & Co, Rahway, NJ, USA) was added to each well and incubated for 4 h. Subsequently, the supernatant was discarded, and 100 µL of dimethyl sulfoxide (DMSO) was added to each well to dissolve the purple formazan crystals formed in viable cells. The absorbance of the dissolved formazan crystal was measured at 570 nm, with 630 nm as the reference wavelength, using a UV-vis spectrophotometer microplate reader (Infinite 200 Pro, Tecan, Männedorf, Switzerland). All data were curated in triplicate, and the cell viability (%) was calculated with the following formula:

$$\mathrm{Cell}\;\mathrm{viability}\;\%=\frac{\mathrm{Absorbance}\;\mathrm{of}\;\mathrm{samples}}{\mathrm{Absorbance}\;\mathrm{of}\;\mathrm{negative}\;\mathrm{control}}\times 100\%$$

### 4.4. Measurement of Nitric Oxide

The NO production of LPS-stimulated BV2 microglia was determined by Griess assay, a common method that measures the amount of nitrite, a relatively stable oxidation product of NO, through the azo-coupling reaction of N-(1-naphthyl)ethylenediamine (NED) and sulphanilamide to visualize NO in pink-red azo dye (Cell Signaling Technology, Danvers, MA, USA). 250 μM of L-NAME (Sigma-Aldrich, St. Louis, Mo, USA) was used as the positive control. First, BV2 microglia were plated in a 96-well plate at a cell density of 6.25 × 10^4^ cells per well and incubated for 24 h. Then, the cells were treated with various concentrations of *K. malesianus* extracts (ethyl acetate, ethanol and methanol extracts) for 2 h, followed by LPS stimulation (1 μg/mL) from *Escherichia coli* (O55:B5, Sigma-Aldrich, St. Louis, Mo, USA) for 24 h. On the third day, 100 µL of supernatant from each well was collected and mixed with an equal amount of Griess reagent in a 96-well plate. The absorbance was measured immediately at 550 nm using a microplate reader. Eight concentrations of nitrite in the range of 0–100 µM were used as the standard. The amount of NO was calculated with reference to the standard curve of nitrite. The most potent extract was selected for subsequent experiments.

### 4.5. Western Blot Analysis

BV2 microglia were plated in a 6-well plate at a cell density of 6.25 × 10^5^ cells per well and incubated for 24 h. The cells were treated with *K. malesianus* methanol extract for 2 h, followed by LPS stimulation (1 μg/mL) for 24 h. Then, the cells were lysed with a lysis buffer (9803; Cell Signaling Technology, Danvers, MA, USA) cocktail supplemented with 1 mM phenylmethylsulphonyl fluoride (PMSF) (Roche diagnostics, Mannheim, Baden-Württemberg, Germany) and protease inhibitor (A32865; Thermo Fisher Scientific, Waltham, MA, USA) for 5 min. The protein lysates were quantified using a Pierce™ BCA protein assay kit (Thermo Fisher Scientific, Waltham, MA, USA). Equal amounts of proteins were separated on SDS-polyacrylamide gels (PAGE) at 120 V for 1.5 h. Then, the protein on the SDS gel was transferred to a nitrocellulose membrane with a semi-dry transfer system (Bio-Rad Laboratories, Hercules, CA, USA) at 25 V for 30 min. The blot was incubated with 5% skim milk for 1 h at room temperature, followed by overnight incubation with primary antibodies of interest (anti-iNOS (1:1000; D6B6S), anti-COX-2 (1:1000; D5H5), anti-phospho-AKT (1:1000; S473), anti-AKT (1:1000; C67E7), anti-phospo-ERK (1:1000; 137F5), anti-ERK (1:1000; T202/Y204), anti-phospo-NF-*κ*B (1:1000;), anti-NF-*κ*B (1:1000;) or anti-β-actin (1:1000; 13E5)) from rabbit (Cell Signaling Technology, Danvers, MA, USA) at 4 °C. The blot was washed with tris-buffered saline with 0.1% Tween 20 (TBST), followed by incubation with goat anti-rabbit horseradish peroxidase-conjugated secondary antibody (1:10,000; Thermo Fisher Scientific, Waltham, MA, USA) for 1 h at room temperature. After washing, the protein blot was incubated with substrate reagent from SuperSignal™ West Femto Maximum Sensitivity Substrate electrochemiluminescene (ECL) (Thermo Fisher Scientific, Waltham, MA, USA), followed by visualization with a gel documentation system (Syngene, GBOX F3, Frederick, MD, USA). The protein expression level was quantified by ImageJ software (1.52v, Wayne Rasband, National Institutes of Health, Bethesda, MD, USA). All data were curated in triplicate. Original Western blot images are shown in Figures S1–S7.

### 4.6. Enzyme-Linked Immunosorbent Assay (ELISA)

The expression of extracellular proinflammatory cytokines (TNF-α and IL-6) of LPS-stimulated BV2 microglia was detected by using an ELISA kit (Quantikine^Ⓡ^ Mouse Immunoassay, R&D System^Ⓡ^, Minneapolis, MN, USA) in a 96-well plate with standard techniques according to the manufacturer’s instruction. Briefly, BV2 microglia were plated in a 6-well plate at a cell density of 6.25 × 10^5^ cells per well and incubated for 24 h. The cells were treated with *K. malesianus* methanol extract for 2 h, followed by LPS stimulation (1 μg/mL) for 24 h. On the third day, the supernatant of the cell culture was collected and centrifuged at 15,000 rpm for 3 min at 4 °C to remove cells. Then, the expression of proinflammatory cytokines was measured based on the respective ELISA kit inserts. Data were curated in triplicate.

### 4.7. Reverse Transcription-Polymerase Chain Reaction (RT-PCR)

The mRNA expression of the proinflammatory mediators and cytokines (iNOS, COX-2, TNF-α, IL-1β and IL-6) of LPS-stimulated BV2 microglia was examined using RT-PCR. BV2 microglia were plated in a T75 flask at a cell density of 3.125 × 10^6^ cells per flask and incubated for 24 h. Then, the cells were treated with *K. malesianus* methanol extract for 2 h, followed by LPS stimulation (1 µg/mL) for 24 h. The cells were harvested and centrifuged at 15,000 rpm for 3 min. Briefly, total RNA was isolated using a ReliaPrep™ RNA cell miniprep system (Promega, Madison, WI, USA) according to manufacturer’s instructions, and the RNA concentrations were determined spectrophotometrically (BioDrop, Cambridge, Cambridgeshire, UK). Then, cDNA was synthesized using a GoScript™ reverse transcription system (Promega, Madison, WI, USA). PCR was then performed using GoTaq^Ⓡ^ qPCR master mix, gene-specific primers and nuclease-free water (Promega, Madison, WI, USA) and run for 40 cycles of amplification. Table 2 shows all the primer sequences. Data were curated in triplicate.

### 4.8. Separation and Analysis of Major Compound(s) Using Liquid Chromatography-Mass Spectrometry (LC-MS)

LC-MS/ESI-MS analysis was performed with an Agilent 1290 Infinity LC system (Agilent Technologies, Santa Clara, CA, USA) coupled with an Agilent 6520 accurate-mass Q-TOF mass spectrometer (Agilent Technologies, Santa Clara, CA, USA) with dual ESI sources operated in positive-ion mode. The MS was operated with the electrospray voltage set to 4000 V, a sheath gas flow of 10 L/min, fragmented voltage of 125 V, gas temperature of 300 °C and nebulizer gas at 45 psig. Chromatographic separation of metabolites was achieved using an Agilent Zorbax Eclipse XDB-C18 (Agilent Technologies, Santa Clara, CA, USA) narrow-bore 2.1 × 150 mm, 3.5 micron (particle size) operated at 25 °C. The column was eluted at a flow rate of 0.5 mL/min with aqueous solvent A: 0.1% formic acid in water and B: 0.1% formic acid in acetonitrile. The chemical structure of all the identified bioactive compounds were visualized using ChemDraw JS Sample Page (version 19.0.0-CDJS-19.0.x+da9bec968, PerkinElmer, Waltham, MA, USA).

### 4.9. Statistical Analysis

Statistical analysis was performed with the Statistical Package for Social Science (SPSS, version 23.0 for IOS, Chicago, IL, USA), and the data were expressed as mean ± standard deviation (SD) of three independent replicates. Levene’s test was used to assess the homogeneity of variance. One-way ANOVA and Duncan’s post hoc multiple comparison test were performed. Statistical differences with *p* ≤ 0.05 were considered significant.

## 5. Conclusions

In conclusion, ethyl acetate, ethanol and methanol extracts of *K. malesianus* inhibited NO production, with methanol extract exhibiting the most potent NO inhibitory activity. Further studies revealed that the *K. malesianus* methanol extract suppressed the expression of proinflammatory mediators and cytokines in LPS-stimulated BV2 microglia via the AKT/NF-*κ*B and ERK pathways. Our findings indicate that *K. malesianus* possesses antineuroinflammatory activity and that prosopinine and eplerenone are the bioactive compounds that contribute to the antineuroinflammatory activity. To expand on the extant knowledge, isolation of both bioactive compounds, prosopinine and eplerenone, and further testing of its antineuroinflammatory activity in an in vivo study are needed.

## Figures and Tables

**Table 1:** Proposed bioactive compounds present in methanol extract of *K. malesianus*.

No	Compound Name	Formula	Chemical Structure	*m/z*	Mass	Bioactivity	References
1.	2,6-Nonadien-1-ol	C_9_H_16_O	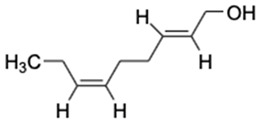	158.154	140.1201	Key aroma-active compound that contributes fresh flavors to black garlic	[29]
2.	Alanyl-Proline	C_8_H_14_N_2_O_3_	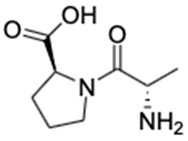	187.1075	186.1003	Inhibitor of human cyclophilin hCyp-18	[30]
3.	Xestoaminol C	C_14_H_31_NO	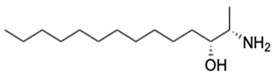	230.2478	229.2405	Antitumor activity, antimicrobial activity and antiparasitic activity	[31,32]
4.	Glutamyl-Proline	C_10_H_16_N_2_O_5_	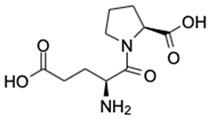	245.1133	244.1059	Antitumor activity	[33]
5.	Prosopinine	C_16_H_33_NO_3_	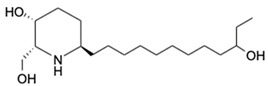	288.2535	287.2463	Anaesthetic activity; antibiotic, analgesic and anti-inflammatory activity	[34,35]
6.	1-Monopalmitin	C_19_H_38_O_4_	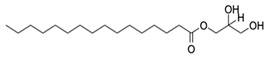	331.284	330.2768	Antitumor activity, antiviral activity	[36,37]
7.	Eplerenone	C_24_H_30_O_6_	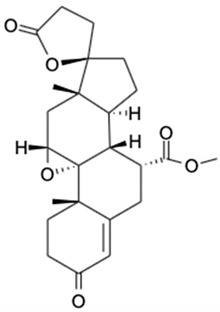	415.2124	414.2048	Reduced mortality and morbidity in patients with acute myocardial infarction; reduced blood pressure; antiinflammatory activity	[38,39,40,41]

**Table 2:** Proinflammatory mediators and cytokines primer sequences.

mRNA Species	Primer Sequence	Reference
iNOS	5′-TTGCCACGGACGAGACGGATAGG-3′ 5′-GGGCACATGCAAGGAAGGGAACTC-3′	[60]
COX-2	5′-TGCTGGTGGAAAAACCTCGT-3′ 5′-GGTGCTCGGCTTCCAGTATT-3′	[60]
TNF-α	5′-GAAAAGCAAGCAGCCAACCA-3′ 5′-CGGATCATGCTTTCTGTGCTC-3′	[61]
IL-1β	5′-GCTGAAAGCTCTCCACCTCA-3′ 5′-AGGCCACAGGTATTTTGTCG-3′	[62]
IL-6	5′-GAGGATACCACTCCCAACAGACC-3′ 5′-AAGTGCATCATCGTTGTTCATACA-3′	[62]
GAPDH	5′-GGAGCGAGACCCCACTAACAT-3′ 5′-GTGAGTTGTCATATTTCTCGTGG-3′	[63]

## Data Availability

Not applicable.

## Supplementary Materials

The following supporting information can be downloaded at: https://www.mdpi.com/article/10.3390/md20080534/s1, Figure S1. Original Western blot images for three repeats of iNOS, COX-2 and beta-actin; Figure S2. Original Western blot images for three repeats of phospho-AKT; Figure S3. Original Western blot images for three repeats of total AKT and beta-actin; Figure S4. Original Western blot images for three repeats of phospho-ERK; Figure S5. Original Western blot images for three repeats of total-ERK and beta-actin; Figure S6. Original Western blot images for three repeats of phospho-NF-*κ*B; Figure S7. Original Western blot images for three repeats of total-NF-*κ*B and beta-actin.
