# Supplementary material for: Carrageenophyte Kappaphycus malesianus Inhibits Microglia-Mediated Neuroinflammation via Suppression of AKT/NF-κB and ERK Signaling Pathways
Source: Mar Drugs. 2022 Aug 20;20(8):534. doi: 10.3390/md20080534 (PMC9410251; doi:10.3390/md20080534)

Figure S1. Original western blot images for three repeats of iNOS, COX-2 and Beta-actin

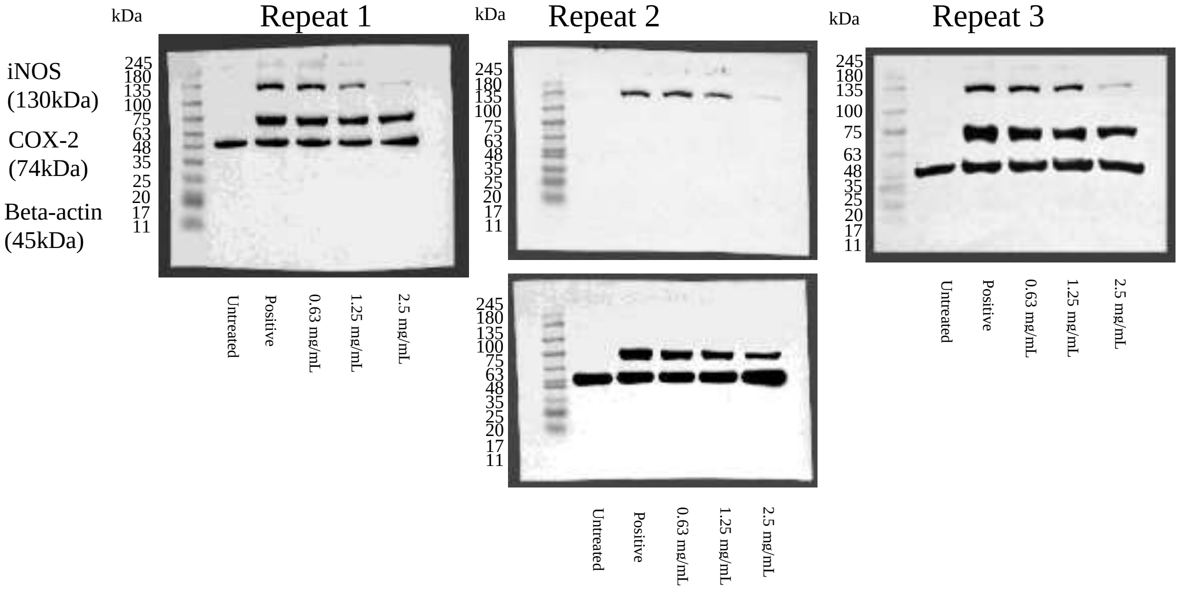

Figure S2. Original western blot images for three repeats of phospho-AKT

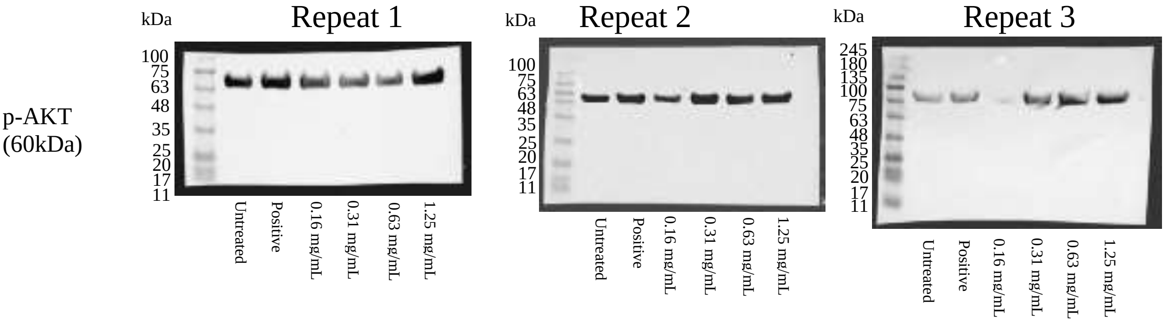

Figure S3. Original western blot images for three repeats of total-AKT and Beta-actin

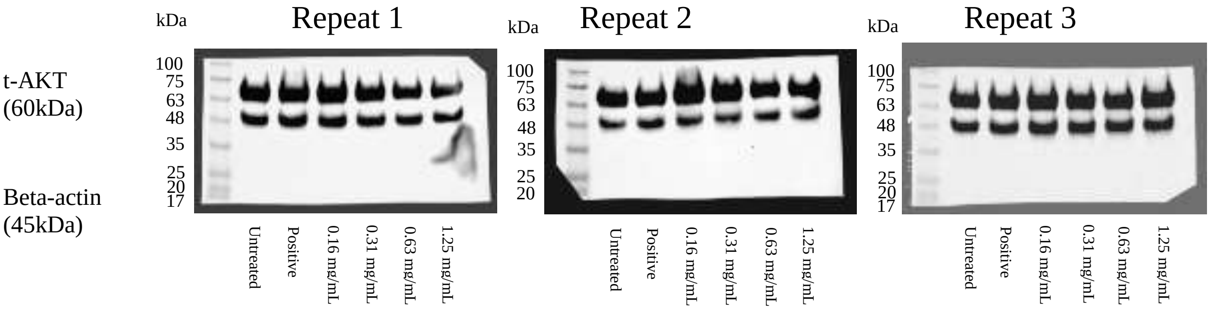

Figure S4. Original western blot images for three repeats of phospho-ERK

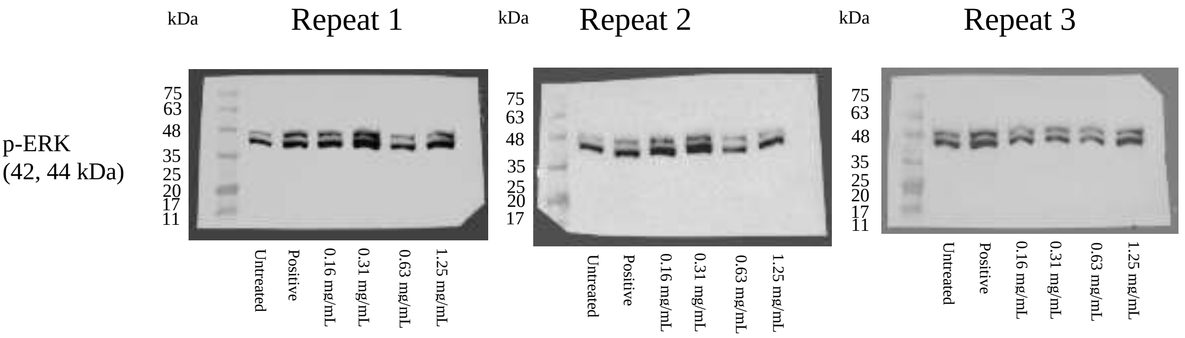

Figure S5. Original western blot images for three repeats of total-ERK and Beta-actin

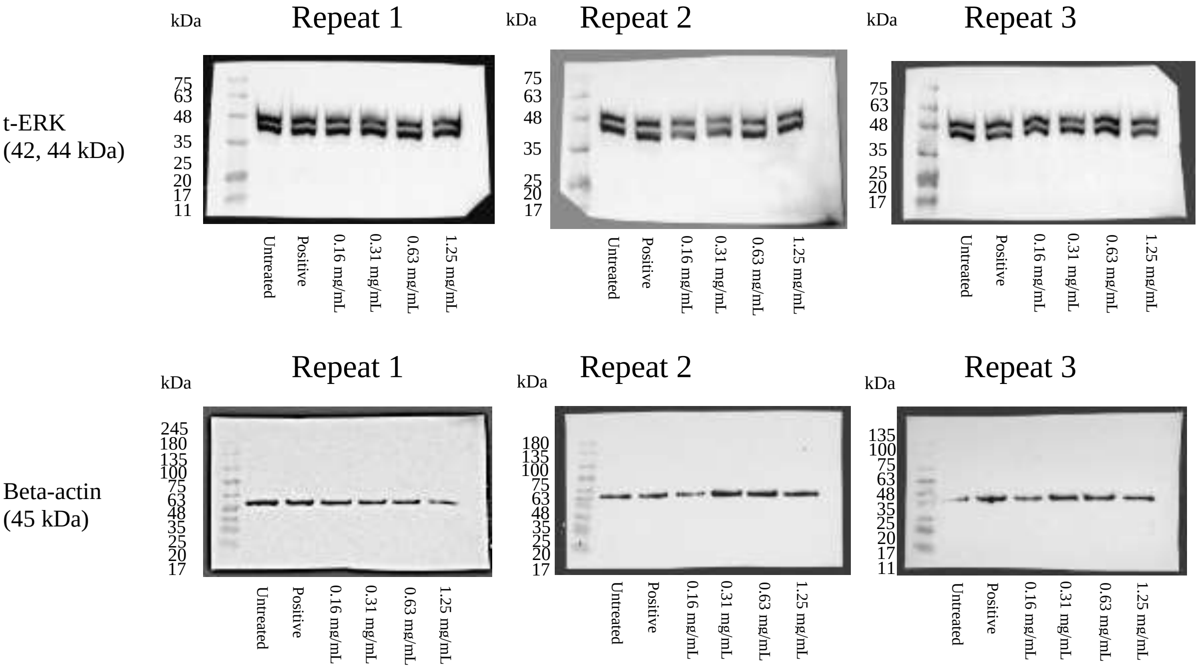

Figure S6. Original western blot images for three repeats of phopho-NF-κB

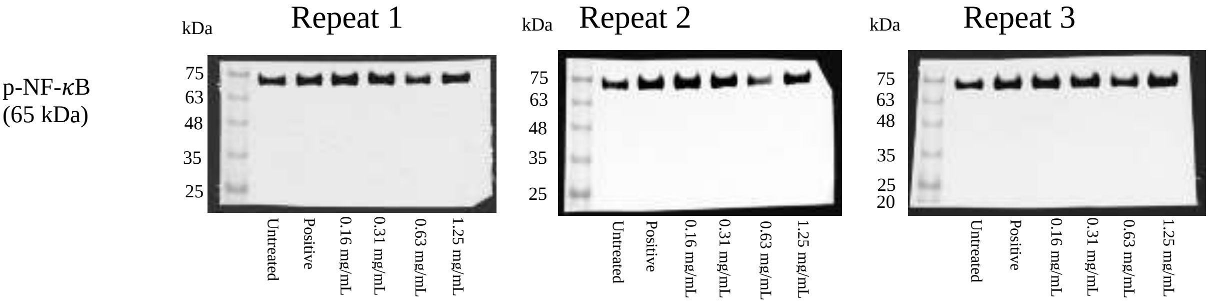

Figure S7. Original western blot images for three repeats of total-NF-κB and Beta-actin

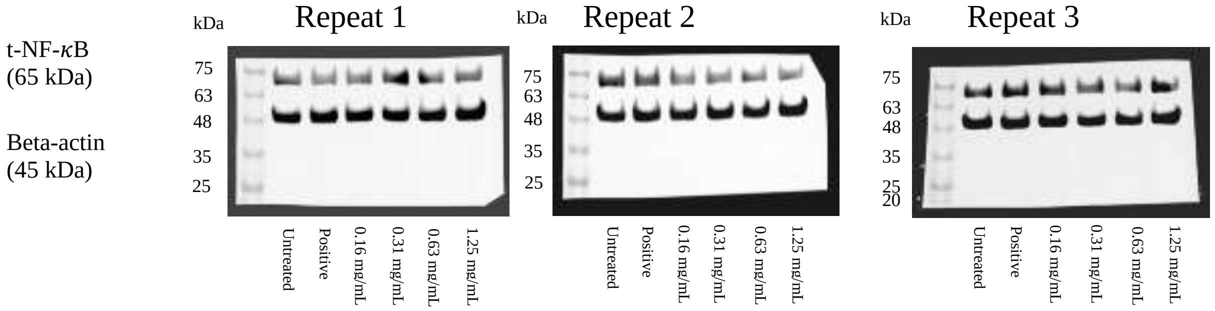

Supplement: Supplementary file 1 [file marinedrugs-20-00534-s001.zip › marinedrugs-1863141-supplementary.pdf]
